# Writing superiority in cued recall

**DOI:** 10.3389/fpsyg.2013.00764

**Published:** 2013-10-18

**Authors:** Carina Fueller, Jens Loescher, Peter Indefrey

**Affiliations:** ^1^Department of Linguistics, Institut für Sprache und Information, Heinrich-Heine-UniversitätDüsseldorf, Germany; ^2^Fachrichtung 4.1 Germanistik, Universität des SaarlandesSaarbrücken, Germany

**Keywords:** paired-associate learning, response modality, writing, cued recall, output modality, input modality

## Abstract

In list learning paradigms with free recall, written recall has been found to be less susceptible to intrusions of related concepts than spoken recall when the list items had been visually presented. This effect has been ascribed to the use of stored orthographic representations from the study phase during written recall (Kellogg, 2001). In other memory retrieval paradigms, by contrast, either better recall for modality-congruent items or an input-independent writing superiority effect have been found (Grabowski, 2005). In a series of four experiments using a paired associate learning paradigm we tested (a) whether output modality effects on verbal recall can be replicated in a paradigm that does not involve the rejection of semantically related intrusion words, (b) whether a possible superior performance for written recall was due to a slower response onset for writing as compared to speaking in immediate recall, and (c) whether the performance in paired associate word recall was correlated with performance in an additional episodic memory recall task. We observed better written recall in the first half of the recall phase, irrespective of the modality in which the material was presented upon encoding. An explanation for this effect based on longer response latencies for writing and hence more time for memory retrieval could be ruled out by showing that the effect persisted in delayed response versions of the task. Although there was some evidence that stored additional episodic information may contribute to the successful retrieval of associate words, this evidence was only found in the immediate response experiments and hence is most likely independent from the observed output modality effect. In sum, our results from a paired associate learning paradigm suggest that superior performance for written vs. spoken recall cannot be (solely) explained in terms of additional access to stored orthographic representations from the encoding phase. Our findings rather suggest a general writing-superiority effect at the time of memory retrieval.

## Introduction

Writing is involved in many experimental tasks, especially in learning and memory research. Yet, output modality is not commonly considered and manipulated as an independent variable although there is evidence both from healthy individuals and aphasic patients that processing differences between written and spoken production exist (cf. Caramazza, 1997; Miceli and Capasso, 1997; Bonin et al., 1998) and, more importantly, may affect the performance in tasks involving memory retrieval (Linton and Brotsky, 1969; Kellogg, 2001; Grabowski, 2005).

One paradigm in which modality differences have been studied in some detail is list recall. Smith and Hunt (1998) presented participants with several lists of related words, each based on an associated concept (e.g., the words *hot, snow, winter, ice, chilly, freeze, shiver*, and *frost* all relate to the concept *COLD*) and asked to write down as many items as they could recall after each list. The associated word was not present in the list, but upon recall tended to appear as a false memory (“intrusion”). Smith and Hunt (1998) found that when lists were presented visually, there were significantly less intrusions. They concluded that list learning is more accurate, when lists are presented visually rather than aurally. Kellogg (2001) replicated the experiment using the same lists but varying the output modality. He confirmed that written recall from visual input was more accurate than from aural input. In spoken recall, however, input modality had no effect. According to Kellogg (2001), the lower proportion of false memories in written recall with visual presentation can be ascribed to the orthographic representation that is encoded in addition to phonological and semantic information when the stimulus word is presented visually. The concurrent availability of the graphemic code serves as an additional source of information in the recall process and helps to reject false memory intrusions. Kellogg (2001) further presumes that only preparing for written output will (re)activate orthographic features, which is why the encoded graphemic code only helps in written, but not in spoken recall even though orthographic encodings are equally available in both conditions. Thus, according to Kellogg (2001) the benefit of writing hinges on the encoding of orthographic information during list learning and is due to a process that is very specific for the list recall task, i.e., rejecting false memories.

Grabowski (2005), in contrast, argues for a general superiority of writing in knowledge recall. In different experiments, he asked students to name or write down as many European countries and capitals as they could think of or to reproduce the names of simple objects that had been presented as pictures in a preceding study phase. In both experiments he found a better performance in writing compared to speaking. Grabowski (2005) argues that writing reduces the cognitive load of maintaining a discourse representation, uses less cognitive resources per time unit, because it is slower, and uses less cognitive resources due to a reduced pressure to produce continuous output. The freed cognitive resources allow for more effective planning processes and information retrieval from long-term memory. In addition to a general writing superiority effect, Grabowski (2005) also obtained evidence for a modality congruence effect in a further experiment testing the recall of verbal material (non-sense sentences) that had been presented either visually or aurally in a study phase. Visually presented sentences were recalled better in writing, aurally presented sentences better in speaking. As in Kellogg's (2001) study, the latter result suggests a use of orthographic information during written recall. Unlike Kellogg's results, however, orthographic information helped to improve correct recall rather than only reduce false memories. Furthermore, auditory episodic information had a similar effect for spoken recall.

There is indeed evidence that episodic information about linguistic stimuli is stored and retained with great accuracy. The presentation modality has been shown to be spontaneously remembered by participants in a number of experiments (Hintzman et al., 1972, 1973; Light et al., 1973; Lehman, 1982). Likewise, studies on printed word perception suggest that episodic memory traces are an integral part of written word perception in that font details or spatial location of a word persist well in memory over time (cf. Jacoby and Hayman, 1987; Goldinger, 1998). The persistence of such episodic information has also been shown for spoken word perception. Detailed auditory information (intonation, pitch, speaker gender) is still accessible after aural presentation of words (cf. Goldinger, 1998) suggesting that episodic information may play an important role in dealing with linguistic stimuli in general.

In sum, the available evidence suggests that there could be several effects of written as compared to spoken output on memory retrieval. A general superiority of writing as a recall modality, independent of written input, an input-dependent effect enhancing written recall of orthographic input, and an effect on the rejection of false memories by using stored orthographic input representations.

One problem with this view is that the three effects do not seem to be additive and hence independent. Kellogg (2001) found no general advantage for written output and only a marginal effect of enhanced correct written recall of orthographic input. Grabowski (2005), likewise, did not find a main effect of written output in his experiment demonstrating the modality congruence effect. It is also unclear whether the representation of orthographic information that seems to underlie the input-dependent effects is just one aspect of the overall episodic trace of study trials and hence on a par with task-irrelevant episodic information (in other words participants just remembered these trials better) or whether these effects are carried specifically by orthographic information.

We conducted a series of four paired-associate learning experiments with semantically unrelated visual or auditory materials and written (typing on a computer keyboard) or spoken recall to help clarify these issues. This experimental paradigm involves study and recall phases not unlike list recall but is unlikely to elicit intrusions of novel, semantically related words (false memories) and hence takes away the need to reject them. Conversely, “intrusions” of *old* words in response to a wrong cue word are a source of error in this paradigm, but such intrusions of words that have actually been presented cannot be rejected based on the lack of an orthographic memory trace. Consequently, if a general writing superiority effect or a modality congruence effect were largely obscured by an exclusive use of orthographic input representation for the rejection of false memories in list recall, one or both of these effects might resurface in our paradigm. Grabowski's (2005) experiment showing a modality congruence effect but no general writing superiority effect also had a study and recall phase with verbal materials. In his paradigm, the need to recall non-sense sentences, such as “Die Maus trägt den Anwalt.” (The mouse carries the lawyer.) verbatim might have promoted a deeper encoding of orthographic and acoustic/phonological full sentence representations, thus boosting the facilitating effect on modality-matched output representations and obscuring a general writing superiority effect, which might be detectable with the simpler materials of our paradigm.

Between our experiments we varied the instruction to respond either immediately or after a delayed response cue. Based on Bonin et al. (1998), we expected immediate spoken responses to be faster than immediate written responses. A delay of 3 s was chosen to ensure that there was ample time for memory retrieval in both response modalities, such that different response latencies could no longer affect recall performance. Hence we expected a potential writing superiority effect in immediate recall to disappear in delayed recall, if it depended on a slower response latency of writing.

Within experiments we additionally varied visual and auditory task-irrelevant episodic information in the study phase and used the recall of this episodic information as a second dependent variable next to item recall. To the extent that item recall may be based on episodic memory retrieval it should be correlated with the recall of task-irrelevant episodic information, such as background color or noise during the study phase. In other words, the correlation of item recall performance with episodic information recall performance would be informative with respect to the degree to which item recall depended on how well a study trial was remembered in general rather than on the recall of orthographic or acoustic/phonological information alone.

## Experiment 1: visual paired-associate-learning with immediate cued recall

### Methods

#### Participants

A total of 48 (32 female) literate native speakers of German with normal or corrected-to-normal vision participated in the experiment.

#### Stimuli

The stimuli consisted of 50 pairs of arbitrarily selected German mid-to-high-frequency words (mean CELEX frequency 53/million). The two words of a pair were semantically and associatively unrelated according to the “*Noun associates for German*” *(NaG)* Database' (Melinger and Weber, 2006). Ten additional word pairs were used for warm-up trials.

#### Procedure

The procedure was a paired-associate learning task consisting of a study phase and a recall phase. All participants were trained and tested individually in a sound attenuated booth. The experiment was implemented with the software Presentation^®^.

In the study phase, participants were presented visually with the word pairs on a computer screen in a sound attenuated booth at a rate of one word pair every 2.9 s. Presentation trials consisted of a fixation cross for 500 milliseconds (ms), followed by two vertically arranged, center-aligned words for 2000 ms, and a blank screen for 400 ms. The word pairs were presented in the same order to all participants. In Study List 1, half of the word pairs were randomly assigned a blue background, the other half a yellow background. The background color assignment to word pairs was reversed for Study List 2. One half of the participants were trained with Study List 1, the other half with Study List 2. The complete set of 60 word pairs was presented twice with a pause after the first presentation. All participants received written instructions to look at the computer screen and to memorize the presented word pairs as well as possible. No details on the later recall phase were given at this point.

The recall phase took place immediately after the study phase had been completed. In each trial, the participants were presented with the upper word of a studied word pair as a cue word and asked to respond with the associated lower word (response word). The recall phase was divided in a spoken recall block and a written recall block with 25 stimuli each, both preceded by 5 warm-up trials. The initial response modality of the recall phase (“start task” writing or speaking) was counterbalanced across participants.

We randomly assigned 25 word pairs to the writing block and 25 word pairs to the speaking block (Test List 1). The assignment was reversed for Test List 2. Half of the participants of each Study List received Test List 1, the other half Test List 2. Thus, across participants, written and spoken recall involved the same word pairs that had been studied in the same order and with equal proportions of the two background colors. The participants received separate written instructions for the two recall blocks.

In both blocks, the cue words were presented in an individually randomized order, preceded by five warm-up trials using the warm-up stimuli from the study phase to become familiar with the procedure. Warm-up trials were excluded from further analysis. In the writing block, responses were typed on a computer keyboard. In the speaking block, responses were spoken into the microphone of a head set (Sennheiser PC 13). Reaction times were taken from first key press in written recall and voice onset time (VOT) in spoken recall. Spoken responses were recorded on an MP3 recorder (Marantz MD620) that also received a 50 ms beep locked to cue word onset. The beep was not audible to the participants. Using PRAAT-software, VOT was measured manually by determining the elapsed time between cue word onset (beep onset) and the subjects' speech onset.

In both blocks, participants completed their responses by pressing the enter key on a keyboard. Participants were allowed to press the enter key without responding when they didn't recall the response word, and to correct themselves. Trials with no, self-corrected, or incorrect responses were discarded as errors.

The enter key press triggered the appearance of the question “*What was the background color*?” on the computer screen. Participants were instructed to respond by pressing either the key [b] for “blue” (German: blau) or [g] for “yellow” (German: gelb) on the keyboard. Key presses triggered the appearance of the question “*How certain are you about the background color*?” on the computer screen, together with a picture showing a scale from 1 (very uncertain) to 5 (totally certain). The participants submitted their confidence ratings by pressing the corresponding keys (1–5) on the keyboard.

### Results

We analyzed the proportions of correct recall of the response word (“item recall”), the proportions of correct recall of the background color (“color recall”), and item recall latencies. Data from one participant were excluded from analysis due to a proportion of correct item recall of less than 3%, corresponding to 1.5 standard deviations (SDs) below the mean of 39.1% correct responses.

#### Item recall

**Figure 1:**
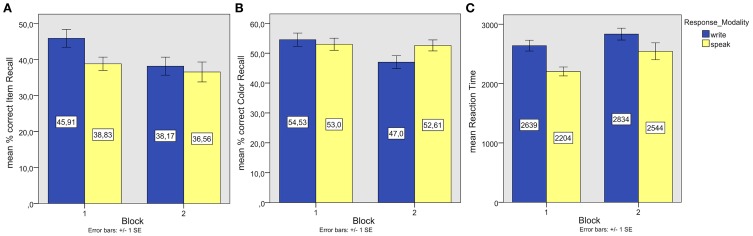
**Experiment 1. (A)** Mean associate word (item) recall performance; **(B)** Mean episodic information (background color) recall performance; **(C)** Mean reaction times of correct associate word recall responses.

Proportions of correct recall (see Figure 1A) were entered into repeated measures ANOVAs of participant and item means with the within-subjects factor Response Modality (write, speak) and the between-subjects factor Start Task (write, speak) in the by-participant analysis and the within-items factors Response Modality (write, speak) and Block (1, 2) in the by-item analysis. Please note that due to the between-subjects manipulation of the order of response modalities a main effect of Block in the by-items analysis corresponds to an interaction of Response Modality and Start Task in the by-participants analysis. Such an interaction may, however, also be the result of performance differences between modalities in only one block and, therefore, warrants *post-hoc* comparisons for interpretation. For easier interpretability of the figures we present all data plotting response modalities against blocks.

Both ANOVAs showed a significant main effect of Response Modality, *F*_1(1, 45)_ = 7.02, *p* = 0.01, partial η^2^ = 0.14; *F*_2(1, 49)_ = 5.43, *p* = 0.02, partial η^2^ = 0.100. A main effect of Block in the by-item analysis, *F*_2(1, 49)_ = 11.170, *p* < 0.00, η^2^ = 0.19, and a Response Modality × Start Task interaction in the by-participant analysis, *F*_1(1, 45)_ = 9.38 *p* = 0.00, η^2^ = 0.17 indicated lower correct item recall in Block 2. Paired samples *t*-tests on item means showed superior written compared to spoken recall in the first block [*t*_(49)_ = 2.97, *p* < 0.01, *r* = 0.39] and no significant difference in the second block (*t* < 1). Kolmogorov–Smirnov-tests and analyzes of skew and kurtosis revealed mild (skew below ±1, kurtosis below ±1.3) deviations from the normality assumption in some combinations of Modality and Block. Non-parametric Wilcoxon Signed-Rank Tests on item means confirmed superior written compared to spoken recall in the first block [*Z* = −2.89, Exact significance (2-sided) *p* = 0.003] and no significant difference in the second block (*p* = 0.378).

To assess a potential influence of the study order of the word pairs on the proportion of correct item recall we also conducted a by-item analysis with an additional between-item factor Study Order (study items 1–10, 11–20, 21–30, 31–40, 41–50). Study Order had no effect and did not interact with the other factors (all *F*s < 1). The effects of Response Modality and Block remained significant.

In 82.9% of the trials with incorrect item recall no response word was given. In 8.2% an incorrect response word was given that had been presented in the study phase. In 8.9% an incorrect response word was given that had not been presented in the study phase.

#### Color recall

One sample *t*-tests on subject and item means of the proportions of correct background color recall (see Figure 1B) showed color recall to be at chance level, except for color recall after writing in the first block [*t*_1(22)_ = 2.08, *p* = 0.049, *r* = 0.41; *t*_2(49)_ = 2.05, *p* = 0.045, *r* = 0.28]. The median confidence rating was 3 for color recall after writing in the first block and 2 in the other conditions.

**Figure 2:**
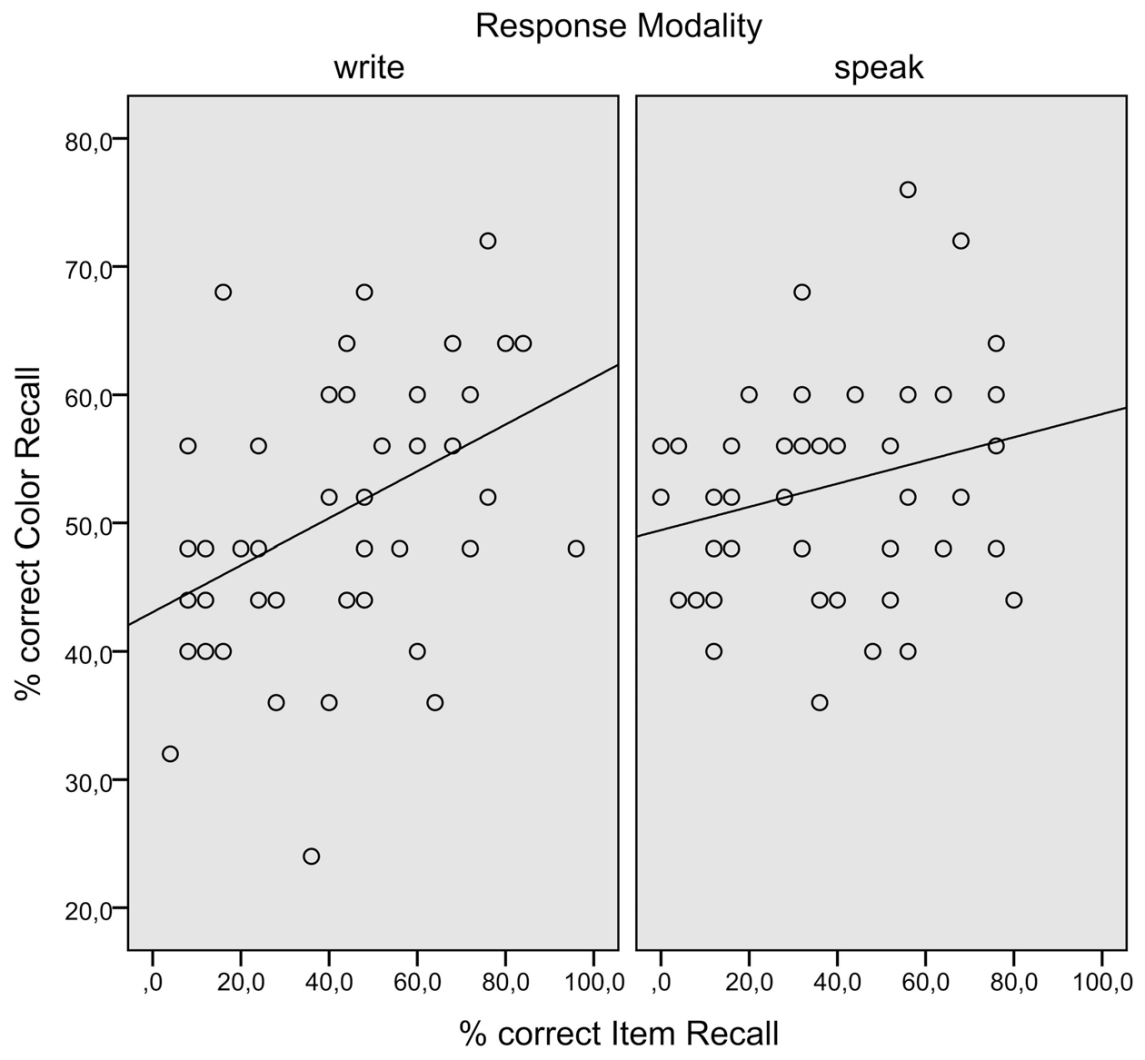
**Experiment 1.** Individual episodic information (background color) recall performances plotted against associate word (item) recall performances.

A positive correlation between color recall and item recall (see Figure 2) reached significance in the written modality, *r* = 0.42, *p* < 0.00, but not in the spoken modality (*r* = 0.25, *p* < 0.09).

#### Reaction times

The data of seven participants, who did not have any correct spoken item recall responses or whose average reaction times were beyond 1.5 SDs (*SD* = 1522 ms) from the mean average RT (3529 ms) of all participants were excluded from further analysis. The reaction times of the remaining participants were cleaned of outliers (RTs beyond 1.5 SDs from the individual means per response modality). Reaction times (see Figure 1C) were entered into repeated measures ANOVAs of participant and item means with the within-subjects factor Response Modality (write, speak) and the between-subjects factor Start Task (write, speak) in the by-participant analysis and the within-items factors Response Modality (write, speak) and Block (1, 2) in the by-item analysis.

The by-participant analysis showed a trend toward a main effect of Response Modality [*F*_1(1, 38)_ = 3.39, *p* = 0.08, partial η^2^ = 0.08] and a significant interaction of Response Modality and Start Task [*F*_1(1, 38)_ = 4.68, *p* = 0.04, partial η^2^ = 0.11]. In the by-item analysis, there were significant main effects of Response Modality [*F*_2(1, 46)_ = 9.09, *p* < 0.00, partial η^2^ = 0.17], indicating longer response latencies in written recall, and Block [*F*_2(1, 46)_ = 6.64, *p* < 0.01, partial η^2^ = 0.13], indicating slower response latencies in Block 2. There was no interaction (*F* < 1).

### Discussion

The first experiment yielded some basic findings, against which the results in the subsequent experiments can be evaluated. Firstly, the memory representations of the studied associations between unrelated words seem to undergo a relatively fast decay as recall proportions were generally lower in the second block. More interestingly, written recall significantly improved the retrieval of visually studied associate words compared to spoken recall, however, this effect was mainly carried by responses in the first block of the recall phase. There was some indication that written recall of the associate word might be supported by the episodic recall of study trials as it showed a correlation with the recall of background color, and background color was recalled above chance and with higher confidence in written recall trials of the first block.

So far, our results are compatible with previous accounts assuming that the recall modality writing helps to reactivate and exploit stored orthographic information from the input, either specifically (Kellogg, 2001) or as the visual instantiation of a modality congruence effect (Grabowski, 2005).

However, as expected, response latencies in writing were considerably slower than response latencies in speaking. Our data, therefore, do not rule out the possibility that better item recall might not be related to the response modality as such but simply due to the extra time available. We addressed this issue in Experiment 2.

## Experiment 2: visual paired-associate learning with delayed cued recall

In Experiment 2, we used an identical study phase but delayed participants' responses in the recall phase by instructing them to respond after they saw a response cue that appeared 3 s after the onset of the cue word. In this way we provided participants with ample time to prepare their responses in both modalities. Our reasoning was that if written recall had been superior to spoken recall in Experiment 1, because the participants had about 400 ms less time for item retrieval in speaking, then the performance difference should disappear when time constraints were removed.

### Methods

#### Participants

Forty-eight (32 female) literate native speakers of German with normal or corrected-to-normal vision who did not participate in Experiment 1 were tested.

#### Stimuli and procedure

Stimuli and study procedure were identical to those used in Experiment 1. The recall phase was identical as well, except for the modification that participants were instructed not to answer until a response cue “?” appeared on the screen. The response cue was presented 3 s after the onset of the written cue word. Trials, in which typing or speaking occurred before the response cue, were discarded.

### Results

As in Experiment 1, we analyzed the proportions of correct item recall, the proportions of correct color recall, and item recall latencies. Data from three participants were excluded from further analysis due to a proportion of correct item recall of less than 12%, corresponding to 1.5 SDs below the mean of 58.1% correct responses.

#### Item recall

**Figure 3:**
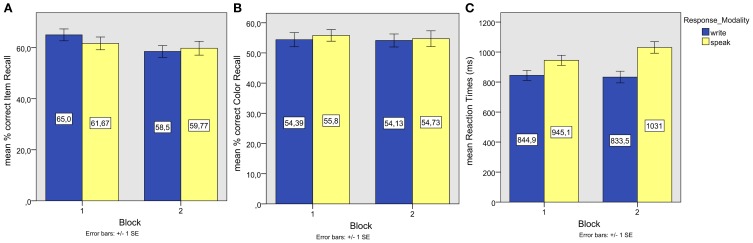
**Experiment 2. (A)** Mean associate word (item) recall performance; **(B)** Mean episodic information (background color) recall performance; **(C)** Mean reaction times of correct associate word recall responses.

Proportions of correct recall (see Figure 3A) were entered into repeated measures ANOVAs of participant and item means with the same factors as in Experiment 1. The by-participant analysis showed no main effect of Response Modality but a significant interaction of Response Modality and Start Task [*F*_1(1, 43)_ = 11.67, *p* = 0.00, partial η^2^ = 0.213]. The by-item ANOVA showed a corresponding significant main effect of Block [*F*_2(1, 49)_ = 6.050, *p* = 0.02, partial η^2^ = 0.11] and no main effect of Response Modality or interaction, indicating that items were recalled more accurately in the first part of the recall phase, independent of the response modality. An additional factor Study Order had no effect (*F* < 1) and did not interact significantly with the other factors (Modality by Block by Study Order: *F* = 1.552, *p* = 0.20, all other *F*s < 1). The effect of Block remained significant. Paired samples *t*-tests on item means showed no significant differences between response modalities in the first or second block (both *p* > 0.1).

In 80.6% of the trials with incorrect item recall no response word was given. In 9.2% an incorrect response word was given that had been presented in the study phase. In 10.2% an incorrect response word was given that had not been presented in the study phase.

#### Color recall

One sample *t*-tests on subject and item means of the proportions of correct background color recall (see Figure 3B) showed color recall to be significantly above chance level after spoken responses in the first block [*t*_1(21)_ = 2.86, *p* = 0.01, *r* = 0.53; *t*_2(49)_ = 3.07, *p* = 0.00, *r* = 0.40]. Proportions of correct background color recall approached significance in the other conditions (all *p* < 0.1). The median confidence rating was 3 in all conditions, except for color recall after speaking in the second block (median = 2).

Unlike experiment 1, there was no significant correlation of background color and associate word recall.

#### Reaction times

The data of four participants, whose average reaction times of correct item recall responses were beyond 1.5 SDs (*SD* = 789 ms) from the mean average RT (1397 ms) of all participants, were removed from further analysis. The reaction times of the remaining participants were cleaned of outliers as in Experiment 1. Reaction times (see Figure 3C) were then entered into ANOVAs of participant and item means with the same factors as in Experiment 1. The by-participants analysis showed no significant effects of Response Modality or Start Task, and no significant interactions (all *F* < 1). The by-items analysis showed a significant effect of Response Modality [*F*_2(1, 49)_ = 5.52, *p* = 0.02, partial η^2^ = 0.10], indicating that spoken responses were slower.

### Discussion

Experiment 2 confirmed a better item recall in the first compared to the second block of the recall phase, but no longer showed a main effect of Response Modality, although there was still a numerically better item recall in writing compared to speaking in the first block. There was also no longer a correlation between item recall and episodic memory recall in writing.

This result suggests that superior written recall in Experiment 1 may indeed have largely been due to the longer response latency of writing. Given a persistent numerical difference in the direction observed in Experiment 1, however, we will postpone a further discussion of this issue until after the experiments testing response modality effects on the recall of aurally presented associate words, to which we now turn.

## Experiment 3: aural paired-associate learning with immediate cued recall

In Experiment 1 we tested for possible effects of output modality on the recall of visually presented word pairs and found that, predominantly in the first block of the recall phase, written recall was superior to spoken recall. Our goal in the third experiment was to determine whether written recall would still affect memory performance positively when no orthographic information from the input would be available during later recall. Persistence of superior written recall would indicate an input-independent, general writing superiority. Superior spoken recall, by contrast, would suggest a modality congruence effect.

### Methods

#### Participants

Forty-eight (24 females) native speakers of German with no history of hearing impairment were tested. None of the participants took part in the previous experiments.

#### Stimuli and procedure

**Figure 4:**
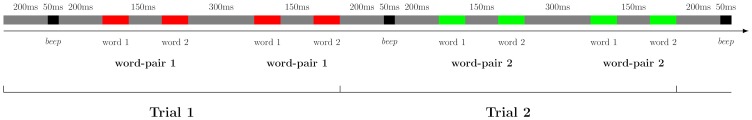
**Aural presentation of word pairs in the study phases of Experiments 3 and 4**.

In the study phase, participants were presented aurally with the word pairs. Study trials (see Figure 4) had an average duration of 4054 s, the exact duration depending on the length of the words. A study trial began with a silent interval of 200 ms duration, followed by 450 Hz warning tone of 50 ms duration. After another silent interval of 200 ms duration a word pair was presented twice consecutively with an average presentation duration of 3604 ms. Between the two words of a pair there was a silent interval of 150 ms duration. Between the two consecutive presentations of a word pair there was a silent interval of 300 ms duration. The stimuli were presented via the earphones of a head set (Sennheiser PC 13) in a sound-attenuated booth. Prior to the study phase, there were three test trials using different stimuli for individual volume adjustment.

The word pairs were recorded from a male and a female speaker. The gender of the speaker was distributed over word pairs corresponding to the background color distribution in the previous experiments.

Whereas the complete set of word pairs had been presented twice in the visual experiments, it was presented three times in this experiment as we expected learning to be more difficult with a sequential aural presentation of word pairs. After each presentation of the set of word pairs there was a short break of self-chosen duration.

The recall procedure was analogous to Experiment 1 with the modification that in the episodic memory task, participants were now instructed to respond by pressing either the key [w] for “female” (German: weiblich) or [m] for “male” (German: männlich) on the keyboard.

### Results

We analyzed the proportions of correct item recall, the proportions of correct recall of the gender of the speaker's voice (“voice recall,” and item recall latencies). Data from seven participants were excluded from analysis due to a proportion of correct item recall of less than 23%, corresponding to 1.5 SDs below the mean of 67.9% correct responses.

#### Item recall

**Figure 5:**
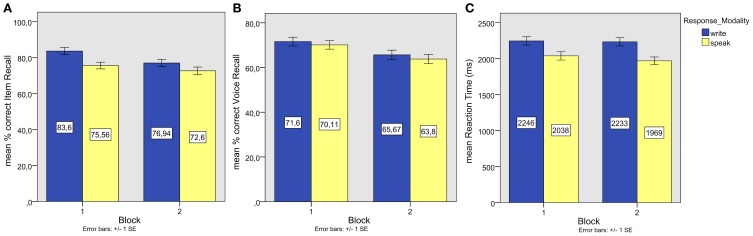
**Experiment 3. (A)** Mean associate word (item) recall performance; **(B)** Mean episodic information (associate word spoken with male or female voice) recall performance; **(C)** Mean reaction times of correct associate word recall responses.

Proportions of correct recall (see Figure 5A) were entered into repeated measures ANOVAs of participant and item means with the same factors as in the previous experiments.

The analyses of subject and item means showed significant main effects of Response Modality [*F*_1(1, 39)_ = 16.896, *p* = 0.00, partial η^2^ = 0.30; *F*_2(1, 49)_ = 12.37, *p* < 0.00, partial η^2^ = 0.20], corresponding to superior written compared to spoken recall. In addition, there was a significant Response Modality × Start Task interaction in the by-participant analysis, *F*_1(1, 39)_ = 9.012, *p* = 0.01, partial η^2^ = 0.188, as well as a significant main effect of Block, *F*_2(1, 49)_ = 6.86, *p* < 0.02, η^2^ = 0.123, but no significant interaction of Response Modality and Block in the by-item analysis (*F*_2_ = 1.31), indicating that the proportion of correct recall was higher in the first compared to the second block for both modalities. An additional factor Study Order had no effect (*F* < 1) and did not interact significantly with the other factors (Modality by Study Order: *F* < 1; Block by Study Order: *F* = 1.762, *p* = 0.15; Modality by Block by Study Order: *F* = 1.494, *p* = 0.22). The effects of Response Modality and Block remained significant.

Paired samples *t*-tests on item means showed superior written compared to spoken recall in the first block [*t*_(49)_ = 3.31, *p* < 0.00, *r* = 0.43]. This difference failed to reach significance in the second block (*p* = 0.07). Kolmogorov–Smirnov-tests and analyses of skew and kurtosis revealed mild (skew below ±1, kurtosis below ±1.3) deviations from the normality assumption in some combinations of Modality and Block. Wilcoxon Signed-Rank Tests on item means confirmed superior written compared to spoken recall in the first block [*Z* = −3.22, Exact significance (2-sided) *p* = 0.001] and no significant difference in the second block (*p* = 0.088).

In 65.0% of the trials with incorrect item recall no response word was given. In 11.5% an incorrect response word was given that had been presented in the study phase. In 23.5% an incorrect response word was given that had not been presented in the study phase.

#### Voice recall

One sample *t*-tests on subject and item means of the proportions of correct background color recall (see Figure 5B) showed voice recall to be significantly above chance level in all conditions (all *p* < 0.05). The median confidence rating was 3 in all conditions, except for voice recall after speaking in the first block (median = 4).

Proportions of correct voice recall were entered into repeated measures ANOVAs of participant and item means with the within-subjects factor Response Modality and the between-subjects factor Start Task in the by-participant analysis and the within-items factors Response Modality and Block in the by-item analysis. There was a significant interaction of Response Modality and Starting Task in the by-subject analysis, *F*_1(1, 39)_ = 9.39, *p* < 0.00, partial η^2^ = 0.194 and a significant main effect of Block in the by-item analysis, *F*_2(1, 49)_ = 13.37, *p* < 0.01, partial η^2^ = 0.214, indicating that voice recall was generally superior in the first block of the recall phase. Response Modality had no significant main effects and did not interact with Block (all *F* < 1).

**Figure 6:**
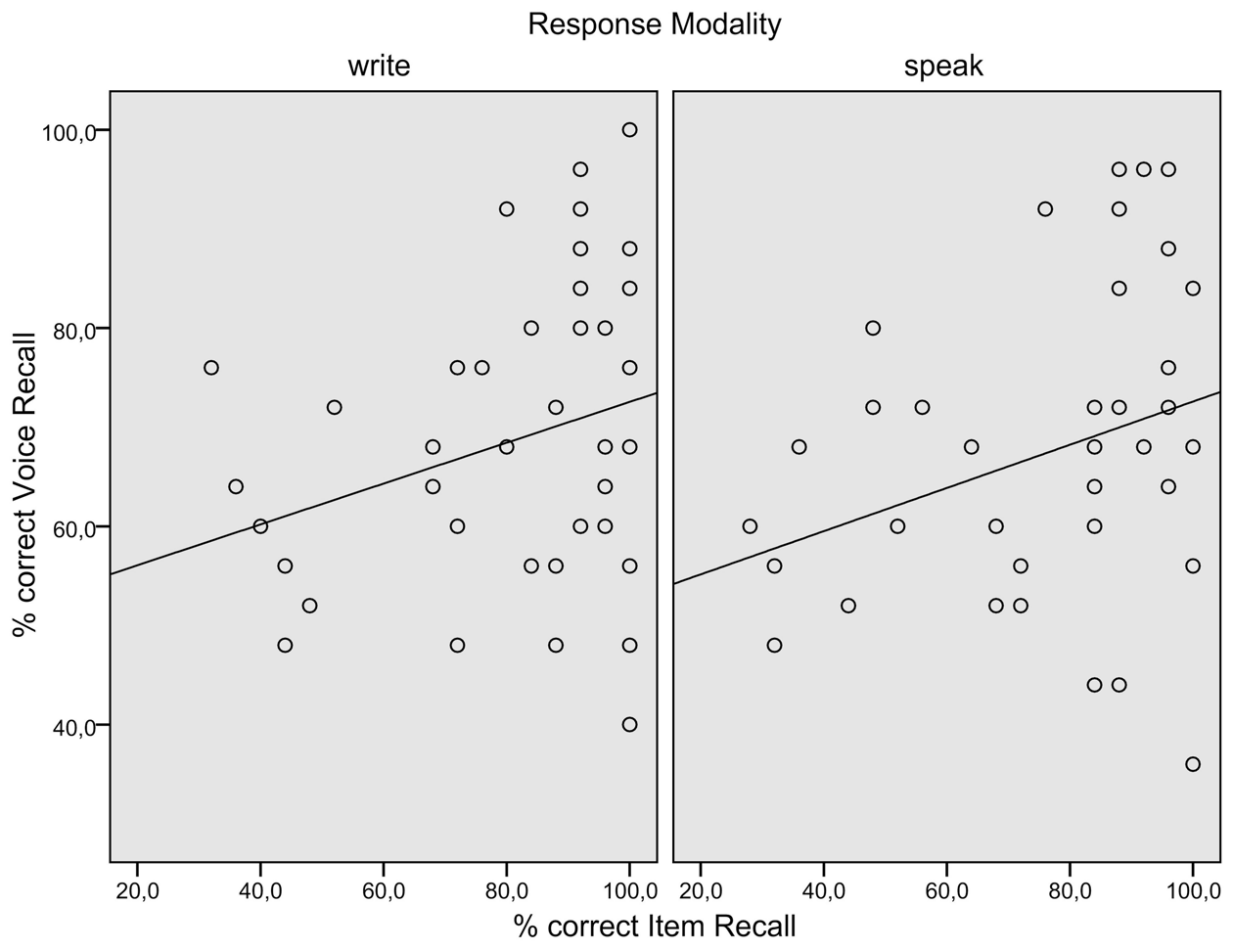
**Experiment 3.** Individual episodic information (associate word spoken with male or female voice) recall performances plotted against associate word (item) recall performances.

Correlations of performances on voice recall and item recall (see Figure 6) reached significance for speaking (*r* = 0.33, *p* = 0.04), but not for writing (*r* = 0.29, *p* = 0.07).

#### Reaction times

The data of four participants, whose average reaction times of correct item recall responses were beyond 1.5 SDs (*SD* = 857 ms) from the mean average RT (2770 ms) of all participants, were removed from further analysis. The reaction times of the remaining participants were cleaned of outliers as in the previous experiments. Reaction times (see Figure 5C) were then entered into ANOVAs of participant and item means with the same factors as in the previous experiments. Both ANOVAs showed a significant main effect of Response Modality [*F*_1(1, 35)_ = 8.87, *p* < 0.01, partial η^2^ = 0.20, *F*_2(1, 49)_ = 17.11, *p* < 0.00, partial η^2^ = 0.259], indicating that written responses were slower. There were no other main effects or interactions (all *F* < 1).

### Discussion

As in Experiment 1, we found written recall to be superior to spoken recall and this difference was more pronounced in the first block. We also replicated a generally better recall performance in the first block. The observed superior written recall of aurally presented associate words is not compatible with a modality congruence effect (Grabowski, 2005), which would have predicted the opposite finding. Our result also rules out that the mechanism shown to underlie a more efficient rejection of false memories in written list recall, namely a re-activation of previously encoded orthographic information during writing (Kellogg, 2001), could be responsible for the writing superiority effect we observed in our task.

By contrast, the combined results of Experiments 1 and 3 are best described as an input-independent writing superiority effect. So far, we have only observed this effect reliably in the two immediate recall experiments that also showed longer onset latencies for writing, suggesting that the main advantage of writing might be that it is a relatively slow manner of language production. As in Experiment 2, we again tested this option by using a delayed recall task in the fourth experiment.

A somewhat surprising finding was the observed significant correlation between spoken item recall and the recall of the gender of the speaker's voice. Taken together with the correlation observed in Experiment 1, this finding might suggest a kind of indirect modality congruence effect, in the sense that episodic information that is congruent with the output modality is more strongly related to item recall in that modality than in the incongruent modality (although without making that modality more effective for item recall). When considering this possibility, however, we found that the visual and the auditory episodic information we used were not entirely comparable. Although both were task-irrelevant, the speaker voice was expressed on the studied word pairs, whereas the background color was not. We, therefore, decided to use in the fourth experiment a different kind of acoustic episodic information that matched the properties of the visual episodic information more closely.

## Experiment 4: aural paired-associate learning with delayed cued recall

Analogous to Experiment 2, this experiment served to clarify whether the writing superiority observed in the previous experiment might have been due to longer written response latencies. A second aim was to exclude a possible effect of the type of episodic information used in Experiment 3. We, therefore, used a different type of acoustic episodic information, presence or absence of background noise, that shared the properties of background color in the visual experiments of being neither task-relevant nor expressed on the word pairs themselves.

### Methods

#### Participants

Forty-eight (24 females) native speakers of German with no history of hearing impairment were tested. None of the participants took part in the previous experiments.

### Procedure

The study phase was identical to Experiment 3, except for two modifications. Firstly, we returned to only two repetitions of the item set as used in Experiments 1 and 2. Contrary to our expectations, the recall proportions for aurally studied word pairs were higher in Experiment 3 than they were in Experiments 1 and 2, and a further increase with delayed responses might have led to unwanted ceiling effects. The second modification was that the stimuli that had been recorded from a female speaker were replaced by recordings of the male speaker with a low level of background noise. Stimuli with background noise were created by mixing the recordings of the male speaker of Experiment 3 with a broad-band white noise using Adobe Audition^®^. The signal to noise ratio (SNR), computed by the difference of the root mean square (RMS) amplitude of the recorded word pair and the RMS-amplitude of the white noise, was at a 15 dB level. This SNR was chosen based on results from speech perception in noise experiments that revealed no significant effects of noise on speech comprehension at this level (Plomp and Mimpen, 1979; Beattie et al., 1997).

The recall procedure was identical to the delayed recall procedure of Experiment 2 with the modification that in the episodic memory task, participants were asked to indicate whether there was a background noise in the recording by pressing [j] for “yes” (German: ja) or [n] for “no” (German: nein).

### Results

We analyzed the proportions of correct item recall, of the proportions of correct recall of the background noise (“noise recall”), and item recall latencies. Data from five participants were excluded from analysis due to a proportion of correct item recall of less than 7%, corresponding to 1.5 SDs below the mean of 50.2% correct responses.

#### Item recall

**Figure 7:**
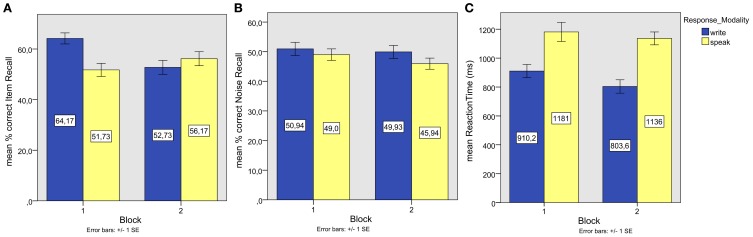
**Experiment 4. (A)** Mean associate word (item) recall performance; **(B)** Mean episodic information (associate word presented with background noise or not) recall performance; **(C)** Mean reaction times of correct associate word recall responses.

Proportions of correct item recall (see Figure 7A) were entered into repeated measures ANOVAs of participant and item means with the same factors as in the previous experiments. There were significant main effects of Response Modality, *F*_1(1, 41)_ = 5.37, *p* = 0.03, partial η^2^ = 0.116, *F*_2(1, 49)_ = 4.990, *p* = 0.03, partial η^2^ = 0.09, as well as a trend toward an interaction of Response Modality and Start Task in the by-participant analysis, *F*_1(1, 41)_ = 3.44, *p* = 0.07, partial η^2^ = 0.077. The by-item analysis yielded a corresponding interaction of Modality and Block [*F*_2(1, 49)_ = 17.42, *p* < 0.00, partial η^2^ = 0.26], indicating superior written recall, when the writing task was performed first. An additional factor Study Order had a significant effect [*F*_2(4, 45)_ = 2.962, *p* < 0.03, partial η^2^ = 0.21] indicating relatively better recall performance for items that were presented in the middle of the study phase. Study Order did not interact significantly with the other factors (Response Modality by Study Order: *F* = 2.280, *p* = 0.075; all other *F*s < 1). The effect of Block was no longer significant (*p* = 0.12). The effect of Response Modality and the interaction between Response Modality and Block remained significant.

Paired samples *t*-tests on item means showed superior written compared to spoken recall in the first block [*t*_(49)_ = 5.01, *p* < 0.00, *r* = 0.58] but not in the second block (*p* > 0.1). Kolmogorov–Smirnov-tests and analyses of skew and kurtosis revealed mild (skew below ±1, kurtosis below ±1.3) deviations from the normality assumption in some combinations of Modality and Block. Wilcoxon Signed-Rank Tests on item means confirmed superior written compared to spoken recall in the first block [*Z* = −4.42, Exact significance (2-sided) *p* = 0.000] and no significant difference in the second block (*p* = 0.383).

In 77.2% of the trials with incorrect item recall no response word was given. In 11.0% an incorrect response word was given that had been presented in the study phase. In 11.8% an incorrect response word was given that had not been presented in the study phase.

#### Noise recall

One sample *t*-tests on subject and item means of the proportions of correct noise recall (see Figure 7B) showed noise recall to be at chance level. The median confidence rating was 3 for noise recall after speaking in the first block and 2 in the other conditions. There was no significant correlation of correct item recall and noise recall.

#### Reaction times

The data of four participants, who did not have any correct spoken item recall responses or whose average reaction times were beyond 1.5 SDs (*SD* = 859 ms) from the mean average RT (1535 ms) of all participants were excluded from further analysis. Reaction times to two items that did not have any correct responses in the spoken condition of the first block were also discarded. The reaction times of the remaining participants were cleaned of outliers as in the previous experiments. Reaction times (see Figure 7C) were then entered into ANOVAs of participant and item means with the same factors as in the previous experiments. Both ANOVAs showed a significant main effect of Response Modality *F*_1(1, 38)_ = 14.66, *p* < 0.00, partial η^2^ = 0.28, *F*_2(1, 47)_ = 34.40, *p* = 0.00, partial η^2^ = 0.42 indicating that written responses were faster. In addition, the by-items ANOVA showed a significant effect of Block [*F*_2(1, 47)_ = 4.49, *p* = 0.04, partial η^2^ = 0.9], indicating slower responses in Block 2.

### Discussion

Somewhat unexpectedly (based on our findings in Experiment 2), this experiment yielded the clearest writing superiority effect and the clearest evidence for this effect being driven by the performance in Block 1 of the recall phase of all four experiments. Given the lack of time constraints for item retrieval due to the use of a delayed response cue and the consistently faster written compared to spoken response onsets in this experiment, we must conclude that writing superiority does not depend on writing being slower than speaking, even if we have no alternative explanation why written item recall failed to be significantly better than spoken recall in the previous delayed recall experiment.

Likewise, superior item recall in writing does not seem to be related to the retrieval of episodic information as this and the two previous experiments showed no correlation between item recall and episodic memory recall in writing. The lack of such a correlation for speaking in this experiment, furthermore, makes any systematic indirect modality congruence effect along the lines suggested in the previous discussion section rather unlikely.

## General discussion

In a series of experiments we addressed the question of the possible relationships between previously reported modality effects on memory retrieval. More specifically, we wanted to know whether a general, input-independent writing superiority effect suggested by Grabowski (2005) would be found in a paired-associate learning paradigm. As explained in the introduction, this paradigm did not share certain design properties of previous experiments by Kellogg (2001) and Grabowski (2005, Experiment IV) that might have favored the effects they found, namely a facilitating effect of previously encoded orthographic representations on the rejection of false memories (Kellogg, 2001) and a modality congruence effect (Grabowski, 2005) but possibly obscured a general writing superiority effect.

We varied input modality between experiments, output modality within subjects, and order of output modalities between subjects, such that half of the participants responded in writing in the first half (block) of the recall phase, the other half in the second block. Across the four experiments we obtained a consistent result: associate words were recalled better in writing than in speaking but only in the first block of the recall phase. Note, however that in Experiment 2 this pattern was found numerically, but did not reach significance. The observation that item recall in writing was better irrespective of the study modality characterizes our result as a general writing superiority effect.

It is important to note that our result does not question the previously reported modality effect reported by Kellogg (2001) or the modality congruence effect reported by Grabowski (2005). The fact that their experiments in contrast to ours did not yield a general writing superiority effect rather confirms that the different modality effects are not independent or additive. The relationship between the different effects rather seems to be such that the more relevant the use of orthographic input representations is for the experimental task at hand the less likely it is to observe a general, input-independent writing superiority effect.

Before discussing possible sources of this effect in detail, it is important to consider whether it might be the result of any constant aspect of our design unintentionally facilitating written item recall. The only aspect that came to our minds in this respect is the use of a visual cue word in the recall phase of all four experiments. However, we rejected the possibility that a visual cue word could have led to superior item recall in all four experiments based on the following considerations of its possible consequences. In Experiments 1 and 2, the visual cue word matched the orthographic format of the corresponding first word of a stimulus word pair in the study phase, whereas this was not the case in Experiments 3 and 4. The orthographic cue might, therefore, have facilitated the recall of the associate word in the first two experiments or hindered it in the last two experiments. There is, however, no evidence of this kind of influence as the mean proportions of item recall were not lower in Experiments 3 and 4 as compared to Experiments 1 and 2. Nonetheless, we refrain from any comparison of the mean proportions of item recall between experiments for this reason and at least two others. Firstly, the item recall performance differed considerably between the participant groups even for experiments 1 and 2, which had the exact same study phases. Secondly, even if we matched the visual and the aural presentation of word pairs in some respects, there was still the main difference between simultaneous visual and sequential aural presentation, which quite plausibly might have affected the strength of the learned association of the word pairs. Importantly, our main results do not hinge on any direct comparison between visual and aural input studies but are based on the differences between written and spoken output *within* experiments. For this comparison, differences between visual and aural input experiments in general or the extent to which the cue word might have differentially facilitated the recall of the study items in particular are not relevant.

In sum, the visual cue word conceivably (although not evident in our data) facilitated the recall of items that had been presented in the same modality but there is no plausible way in which it might have facilitated the recall of items that had been presented aurally in the written compared to the spoken output modality.

### Possible sources of a general writing superiority effect

As described in the introduction, Grabowski (2005) discusses three properties of written language production that might reduce the recruitment of cognitive resources, which can then be used for other processes to improve memory retrieval performance. Two of these properties, reduced constraints on the pacing of language production and reduced effort for discourse representation, do not seem to be relevant in our paradigm. Language production was not internally paced in our paradigm as it only required single word responses that were externally paced. Although writing may reduce the memory load for maintaining a discourse representation, this property cannot have been helpful in our task, because the written responses could not have served as an external storage as they disappeared before the next trial. One reviewer pointed out a plausible further option: When writing was the response modality, orthographic memory traces of written responses might have helped to prevent erroneous intrusions of previous responses. To assess this possibility, we analyzed the proportions of erroneous repeated responses in our data. Across all experiments, 74 erroneous response words (corresponding to 9.3% of all erroneous response words and 2.0% of all errors) had been given as responses in previous trials. In the majority of these cases (71.6%), the word had not been given as a response in the same modality but in the preceding block. Erroneous within-block response repetitions occurred only 13 times in Block 1 and 8 times in Block 2. Thus, response repetitions in two different modalities outnumbered response repetition in the same modality by more than 2.5 to 1. Although there are obviously alternative explanations (e.g., response repetitions in two different modalities occurred by necessity in different blocks, so that the number of intervening items was higher and more time had passed), this asymmetry may indeed suggest that memory traces of previous responses given in the same modality could be used to prevent their erroneous intrusion. Crucially, if our finding of a superior performance in writing in the first block was due to orthographic memory traces being more effective in preventing intrusions of previous response words, one would expect a much lower proportion of written compared to spoken erroneous repeated response words in the first block. Instead, we found an approximately balanced distribution (6 written and 7 spoken erroneous repeated response words). Given that the number of errors was generally lower when writing was the response modality in Block 1 (784 errors in writing, 986 errors in speaking), the proportion of errors due to erroneous repetition of a preceding response words was even slightly higher for writing (0.8%) compared to speaking (0.7%). It thus seems that in our data orthographic traces were no more effective in rejecting intrusions from previous responses in writing than acoustic or phonological traces were in speaking, so that the observed writing superiority cannot be explained by such a mechanism. Still, our data cannot completely rule out an alternative suggestion by this reviewer, namely that orthographic traces are effective in rejecting intrusions of previous responses that do not surface as overt responses but nonetheless interfere with correct recall so that the proportion of erroneous null responses increases. This assumption would not only predict that written recall is superior in the first block but could also explain why this effect might be neutralized in the second block: Even for spoken responses, orthographic traces from the preceding (writing) block could help reject interfering intrusions. Although we find this suggestion very interesting, we are nonetheless hesitant to adopt it. There is no direct evidence for it in our data and the assumption that orthographic traces are only effective in rejecting covert intrusions but do not reduce the proportion of overt intrusions seems somewhat problematic.

The third property, reduced use of cognitive resources per time unit due to slower language production in writing, could have explained a writing superiority in our experiments with immediate responses (Experiments 1 and 3). In both experiments, writing onset was indeed several hundred milliseconds slower than voice onset. To assess, whether this latency difference was responsible for the modality difference in item recall performance, we used a delayed response variant of our task in Experiments 2 and 4. The delay was meant to take away any time constraints for memory retrieval, thus making a potentially persisting delayed response latency difference with slower responses for writing (Bonin et al., 1998) irrelevant. In fact, the delayed response task even caused a reversal of the latency difference between output modalities. Taking the latency of spoken responses as a common baseline, typing in our study was thus faster than handwriting in the study of Bonin et al. (1998). Assuming that reaction times in a delayed response task mainly reflect the processing of response execution because previous processing steps are completed before the response cue, this result suggests considerably reduced motor planning requirements for typing compared to handwriting.

Crucially, the superiority of written item recall was not affected by the delay as such or shorter writing compared to voice onset latencies after the delay, thus ruling out a reduced use of cognitive resources per time unit as a potential source of the effect.

Given that previously suggested reasons for a general writing superiority effect do not seem to apply in our study, we must attempt to constrain possible explanations based on the properties of the effect as shown in our data. Most importantly, the effect was only found in the first block of the recall phase. This observation can mean two things: either writing only facilitates the retrieval of a fast decaying memory trace that is no longer available after the first half of the recall phase or written recall is only superior when it is not preceded by spoken recall.

A memory trace that showed some evidence for decay between Block 1 and 2 was episodic information from the study phase. If this kind of information underlay better item recall in writing, one would expect item recall performance to be highly correlated with performance in the forced choice task testing the recall of episodic information. We found small but significant correlations in both immediate recall experiments but not the delayed recall experiments. These correlations suggest that, not surprisingly, a more vivid recollection of the corresponding study episode including task-irrelevant aspects indeed helped the immediate recall of an item to a small extent explaining 17% of the variance of written item recall in Experiment 1 and 13% of the variance of spoken item recall in Experiment 3. Crucially, however, there was no indication that the observed general superiority of written recall might be related to a better recollection of study episodes. There was no significant correlation in the delayed response experiments, and in Experiment 3 the stronger correlation was found for spoken recall whereas written recall showed the superior performance.

The learned associations also showed decay between the first and the second half of the recall phase. Based on our data, we can, therefore, not exclude that writing somehow selectively improves the recall of very recent associations. Note, however that the nature of Grabowski's (2005, Exp.I) retrieval task makes this option rather unlikely. In this task, he observed a writing superiority effect for the retrieval of geographic knowledge from long-term memory, for which the decay rate is on the order of decades rather than milliseconds.

By exclusion, we must conclude that a general writing superiority effect only appears when writing is not preceded by speaking as a response modality. An area, in which such a dependence on the preceding task context is well-known, is bilingual language processing. A preceding language context can determine the general monolingual or bilingual language “mode” (Grosjean, 1982) of a speaker or listener, and in this way affect the processing of subsequent language input or output. By analogy, writing and speaking may not only be seen as response modalities but also as response “modes,” a kind of mind set that may influence how a particular task such as item recall is approached and performed. If recall starts with writing, certain properties of the “writing mode”—possibly as general as taking the task more seriously—may determine the performance until an arguably more dominant “speaking mode” takes over in the speaking phase. Starting in the “speaking mode” may, however mean that the speaking mode dominates the approach to the task throughout the entire recall phase. This kind of asymmetry is again comparable to bilinguals first receiving input in only one language and then mixed language input. They would be in a monolingual mode until the mixed language input begins. With the reversed order of language inputs, however, they would be in a bilingual mode for the entire period.

## Conclusions

Item recall in paired-associate learning shows a writing superiority effect for both visually and aurally studied items. Superior performance in writing, however, seems to depend on writing being the first output task in the recall phase. Having excluded other explanations as unlikely, the best account for a general writing superiority effect seems to be a certain mind set associated with writing. The positive influence of such a “writing mode” on item recall could be of a quite general nature, for example, taking a task that involves writing more seriously than a task involving speaking.

### Conflict of interest statement

The authors declare that the research was conducted in the absence of any commercial or financial relationships that could be construed as a potential conflict of interest.

## References

[B1] Beattie R. C. Barr T. Roup C. (1997). Normal and hearing-impaired word recognition scores for monosyllabic words in quiet and noise. Br. J. Audiol. 31, 153–164 10.3109/03005364000000018 9276098

[B2] Bonin P. Fayol M. Gombert J. E. (1998). An experimental study of lexical access in the writing and naming of isolated words. Int. J. Psychol. 33, 269–286 10.1080/002075998400312

[B3] Caramazza A. (1997). How many levels of processing are there in lexical access? Cogn. Neuropsychol. 14, 177–208 10.1080/026432997381664

[B4] Goldinger S. D. (1998). Echoes of echoes? An episodic theory of lexical access. Psychol. Rev. 105, 251–279 10.1037/0033-295X.105.2.251 9577239

[B5] Grabowski J. (2005). Der Schriftlichkeits-überlegenheitseffekt: Sprachproduktionsprozesse bei der verbalen Wissensdiagnose. Z. Psychol. 213, 193–204 10.1026/0044-3409.213.4.1939577239

[B6] Grosjean F. (1982). Life with Two Languages: An Introduction to Bilingualism. Cambridge, MA: Harvard University Press

[B7] Hintzman D. L. Block R. Inskeep N. (1972). Memory for mode of input. J. Verbal Learn. Verbal Behav. 11, 741–749 10.1016/S0022-5371(72)80008-2

[B8] Hintzman D. L. Block R. Summers J. (1973). Modality tags and memory for repetitions: Locus of the spacing effect. J. Verbal Learn. Verbal Behav. 12, 229–238 10.1016/S0022-5371(73)80013-1

[B9] Jacoby L. Hayman C. (1987). Specific visual transfer in word identification. J. Exp. Psychol. Learn. Mem. Cogn. 13, 456–463 10.1037/0278-7393.13.3.456

[B10] Kellogg R. T. (2001). Presentation modality and mode of recall in verbal false memory. J. Exp. Psychol. Learn. Mem. Cogn. 27, 913–919 10.1037/0278-7393.27.4.913 11486924

[B11] Lehman E. B. (1982). Memory for modality: evidence for an automatic process. Mem. Cogn. 10, 554–564 10.3758/BF0320243824001008

[B12] Light L. L. Stansbury C. Rubin C. Linde S. (1973). Memory for modality of presentation: within-modality discrimination. Mem. Cogn. 1, 395–400 10.3758/BF03198126 24214575

[B13] Linton M. L. Brotsky S. J. (1969). Administrative procedures in the word association test: method of stimulation and method of esponding (oral vs. written). J. Verbal Learn. Verbal Behav. 8, 123–128 10.1016/S0022-5371(69)80021-6

[B14] Melinger A. Weber A. (2006). Database of Noun Associations for German (NAG). Available online at: http://www.coli.uni-saarland.de/projects/nag/

[B15] Miceli G. Capasso R. (1997). Semantic errors as neuropsychological evidence for the independence and the interaction of orthographic and phonological word forms. Lang. Cogn. Process. 12, 733–764 10.1080/016909697386673

[B16] Plomp R. Mimpen A. (1979). Speech-reception threshold for sentences as a function of age and noise level. J. Acoust. Soc. Am. 66, 1333–1342 10.1121/1.383554 500971

[B17] Smith R. E. Hunt R. R. (1998). Presentation modality affects false memory. Psychon. Bull. Rev. 5, 710–715 10.3758/BF0320885021261425

